# Poor Sleep in Patients with Multiple Sclerosis

**DOI:** 10.1371/journal.pone.0049996

**Published:** 2012-11-14

**Authors:** Hanne Marie Bøe Lunde, Tommy F. Aae, William Indrevåg, Jan Aarseth, Bjørn Bjorvatn, Kjell-Morten Myhr, Lars Bø

**Affiliations:** 1 The Norwegian Multiple Sclerosis Competence Centre, Department of Neurology, Haukeland University Hospital, Bergen, Norway; 2 KG Jebsen Centre for MS-Research, Department of Clinical Medicine, University of Bergen, Bergen, Norway; 3 Norwegian Competence Center for Sleep Disorders, Haukeland University Hospital, Bergen, Norway; 4 Department of Public Health and Primary Health Care, University of Bergen, Bergen, Norway; Charité University Medicine Berlin, Germany

## Abstract

**Background:**

Poor sleep is a frequent symptom in patients with multiple sclerosis (MS). Sleep may be influenced by MS-related symptoms and adverse effects from immunotherapy and symptomatic medications. We aimed to study the prevalence of poor sleep and the influence of socio-demographic and clinical factors on sleep quality in MS- patients.

**Methods:**

A total of 90 MS patients and 108 sex-and age- matched controls were included in a questionnaire survey. Sleep complaints were evaluated by Pittsburgh Sleep Quality Index (PSQI) and a global PSQI score was used to separate good sleepers (≤5) from poor sleepers (>5). Excessive daytime sleepiness, the use of immunotherapy and antidepressant drugs, symptoms of pain, depression, fatigue and MS-specific health related quality of life were registered. Results were compared between patients and controls and between good and poor sleepers among MS patients.

**Results:**

MS patients reported a higher mean global PSQI score than controls (8.6 vs. 6.3, p = 0.001), and 67.1% of the MS patients compared to 43.9% of the controls (p = 0.002) were poor sleepers. Pain (p = 0.02), fatigue (p = 0.001), depression (p = 0.01) and female gender (p = 0.04) were associated with sleep disturbance. Multivariate analyses showed that female gender (p = 0.02), use of immunotherapy (p = 005) and a high psychological burden of MS (p = 0.001) were associated with poor sleep among MS patients.

**Conclusions:**

Poor sleep is common in patients with MS. Early identification and treatment of modifiable risk factors may improve sleep and quality of life in MS.

## Introduction

Patients with multiple sclerosis (MS) frequently report poor sleep, and sleep disorders are more common in MS patients compared to healthy controls [1]. Causes of poor sleep in MS are likely multifactorial, and potential triggers may relate to adverse effects from immunotherapy and symptomatic medications and to MS-associated symptoms, such as pain and fatigue [2]. Certain symptoms related to MS, may lead to exacerbation of others if left untreated [3]. Patients suffering from a sleep disturbance have an increased risk of developing co-morbid conditions like heart disease, obesity and diabetes that may have a profound impact on long-term health [4], [5]. In order to improve sleep and possibly reduce long-term health consequences of poor sleep in MS, identification of modifying risk factors of poor sleep is needed. Therefore we aimed to study the prevalence of sleep disturbance in MS, compared to the healthy Norwegian population. Further, among MS-patients, we aimed to identify possible socio-demographic and clinical factors associated with the risk of being a poor sleeper. Two prior studies have described the socio-demographic and clinical factors associated with poor sleep, both lacking a control group for comparisons [1], [6]. To our knowledge, a case-control study looking at the socio-demographic and clinical associations with poor sleep in MS, has not been published previously.

## Materials and Methods

### Ethics statement

The study was based on written informed consent, and approved by the Regional Committee for Medical and Health Research Ethics of Western Norway.

### Patients and controls

We aimed to include patients' with well-established disease. All 140 patients diagnosed with MS from 1993–1998 at the Department of Neurology, Haukeland University Hospital, Bergen, Norway were invited to participate in the study. All MS-patients fulfilling the diagnostic criteria of Poser were included, independent of subtype. The patients were identified though hospital records and recruited at the Department of Neurology, Haukeland University Hospital. A total of 280 age- and gender-matched controls were drawn from the Norwegian National Population Registry. There were no exclusion criteria. The data were collected by use of validated self-administered questionnaires and returned by mail in pre-stamped addressed return envelopes. All participants received the same set of study information and questionnaires, except for the MSIS-29 questionnaire, specifically designed for MS patients. A reminder was sent to each non-respondent participant via the mail two months after the initial questionnaires were mailed out. The collected data from the questionnaires were registered and entered in a database that was carefully double and triple checked for any mistakes in data entry.

### Variables

Age, gender, marital status and the use of medication were registered. The regular medications were recorded and grouped into three classes: antidepressant drugs, immunotherapy and others. The questionnaires used are all validated, and all but the VAS pain scale are also validated in Norwegian [7]–[11].

Pittsburgh Sleep Quality Index (PSQI) was included to measure sleep and identify sleep complaints during the previous month [12]. It has several clinical and research applications and can be used to separate “good” from “poor” sleepers, screen for night time sleep disturbances and to monitor the progression of sleep disorders [13], [14]. However, it cannot be used to diagnose specific sleep disorders and does not provide information on sleep stages or sleep architecture that can be measured by polysomnography (PSG) [15]. It consists of 19 self- rated questionnaires and 5 additional questionnaires for bed partner.

It provides a global score of sleep on a scale from 1 to 21, with higher scores indicating more sleep complaints. A global PSQI score >5 is shown to have a diagnostic sensitivity of 89.6% and a specificity of 86.5% and was used to separate good sleepers (≤5) from poor sleepers (>5) [12]. Test-retest reliability has shown consistency over time [16].MS-specific health related quality of life was recorded by the Multiple Sclerosis Impact Scale (MSIS-29), that includes the physical (20 items) and psychological (9 items) impact of MS. High scores on MSIS-29 indicate greater impact of MS. Naturally, only MS patients answered these questions, and associations to poor sleep was analysed separately for the physiological and psychological subscales of MSIS-29 (Hobert J) [17].

Excessive daytime sleepiness was registered by the Epworth sleepiness scale (ESS), that is a validated and widely used self-administered questionnaire used to investigate excessive daytime sleepiness/hypersomnia [18]. It can be used as a screening test for excessive sleepiness or longitudinally to follow a patient's response to an intervention. It measures the proneness to fall asleep or doze off in eight specific situations. An ESS score above 10 is regarded as an indicator of excessive sleepiness.

We assessed acute pain by using the validated Visual Analogue Scale for pain assessment. The VAS pain scale consists of a 10 cm (3.94 inches) line with one end labelled “no pain” and the other end labelled “worst imaginable pain ever”. The participants were asked to mark the line at the point that best described the pain intensity. For qualitative analysis we separated the data in two groups “no pain (0–1 points)” and “pain (>1 point) [19]. The presence of pain at the time of completing the questionnaire was registered as present or not [20].

Fatigue was registered by the Fatigue Questionnaire (FQ) that is a validated questionnaire used to assess fatigue severity. It is an 11 item questionnaire used to measure physical and mental fatigue during the previous month (scale 0–3) with a cut off score at 4 considered indicative of excessive fatigue [21].

Symptoms of depression were screened by the Beck Depression Inventory (BDI–II) that includes 21-question self-reporting multiple choice questions. The patients BDI score was categorized as not depressed (score 0–19) and depressed (score>20) [11], [22].

### Statistical analysis

Socio-demographic variables (age, gender, and marital status), medication, and clinical characteristics as well as the seven components of the PSQI questionnaire were compared between patients and controls. Among MS patients, the same variables in addition to health related quality of life (MSIS-29) was compared between good- and poor sleepers.

The Fischer Exact Test was applied to compare groups for nominal variables. The Mantel-Haenszel test was used for ordinal variables and independent two sample t- test was applied for continuous variables. Normal distribution and equality of variance was checked before applying the parametric t-test. Crude p-values are reported.

Independent two sample t- test was applied for continuous variables. Normal distribution and equality of variance was checked before applying the parametric t-test. Crude p-values are reported.

An analysis was performed to identify factors associated with poor sleep. Logistic regression was performed to estimate odds ratios, first in univariate models until a multivariate model was fitted. Variables with univariate *P*<0.25 were selected as candidates in the multivariate analysis following a model-building strategy as described by Hosmer & Lemeshow [23].

Data analyses were carried out using SPSS software for Windows (version 15.0, SPSS, Chicago IL), p-values <0.05 were considered statistically significant.

## Results

### Comparisons of patients and controls

A total of 90 MS patients (64.3%) and 108 (38.6%) control persons responded to the invitation to participate in the survey. Socio-demographic characteristics and the use of antidepressant drugs did not differ significantly among patients and controls (Table 1). The average age of the patients were 45±10.4 and the controls were 44.3±10.2 (p = 0.65). Of the patients, 41(45.6%) were men and 49 (54.4%) were women, and 45(41.7%) men and 63(58.3%) women made up the control group (p = 0.667) (Table 1). The mean score of excessive daytime sleepiness (ESS) did not differ between patients and controls and mean values were below cut-off for excessive sleepiness (data not shown). Pain (p = 0.01), fatigue (p = 0.004) and depression (p = 0.02) were reported more frequently among patients than controls. Thirty-five (42.7%) patients received immunotherapy (interferon beta (n = 24), glatiramer acetate (n = 7), natalizumab (n = 2)) (Table 1). MS patients had a significantly higher mean global PSQI score (8.6±4.6) compared to controls (6.3±4.1), indicating a poorer sleep quality (p = 0.001) (Table 2). PSQI sleep onset latency was significantly higher (1.4±1.1) among patients than controls (1.1±1.1) (0.04) (Table 2). The proportion of MS patients (67.1%) that reported poor quality of sleep was significantly higher than the controls (43.9%) (p = 0.002).

**Table 1:** Clinical and socio-demographic parameters among patients and controls.

Variable	Patients	Controls	p-value
	n = 90*	n = 108**	
			
Age, yrs (mean±SD)	45.0±10.4	44.3±10.2	0.65
Gender (female; %)	54.4	58.3	0.67
Married (%)	58.5	66.7	0.29
Antidepressant drugs (%)	9.8	3.7	0.08
Immunotherapy (%)	42.7	1.9	<0.001
Excessive daytime sleepiness (ESS) (%)	33.8	22.6	0.10
Pain (VAS) (%)	38.8	21.3	0.01
Fatigue (FQ) (%)	35.5	15.8	0.004
Depression (BDI) (%)	15.8	4.2	0.02
MSIS-29 physiological disease severity	27.4±24.7	NA	-
MSIS-29 psychological disease severity	28.1±25.1	NA	-

ESS = Epworth Sleepiness Scale; FQ = Fatigue Questionnaire; BDI = Beck. Depression Inventory; MSIS-29 = Multiple Sclerosis Impact Scale.

* Number of patients range from 76–90;

** Number of controls range from 96–108. Immunotherapy (IFNB, glatiramer acetate, natalizumab).

**Table 2:** Pittsburgh Sleep Quality Index (PSQI) scores among patients and controls.

Variable*	Patients	Controls	*p-value*
	n = 90**	n = 108***	
PSQI global score	8.6±4.6	6.3±4.1	0.001
PSQI sleep quality	1.1±0.7	0.9±0.8	0.03
PSQI sleep onset latency	1.4±1.1	1.1±1.1	0.04
PSQI sleep duration	0.9±0.8	0.8±0.7	0.39
PSQI sleep efficiency	0.7±1.0	0.5±0.8	0.10
PSQI sleep disturbance	1.4±0.6	1.3±0.6	0.33
PSQI hypnotic drugs	0.7±1.2	0.3±0.8	0.005
PSQI daytime dysfunction	1.3±0.8	0.8±0.7	<0.001

* Data are displayed as mean standard deviation;

** Number of patients range from 76–90;

*** Number of controls range from 96–108.

### Risk factors associated with poor sleep among patients with MS

More women (78.0%) than men (53.1%) suffered from poor sleep among the MS patients (p = 0.04). Further, pain (p = 0.02) and fatigue (p = 0.001) were associated with higher risk for being a poor sleeper (Table 3). All patients classified with moderate depression (p = 0.01) and all patients on antidepressant medications suffered from poor sleep (p = 0.03). Excessive daytime sleepiness was not significantly associated with poor sleep among patients (p = 0.12) (Table 3). Higher score on mean MSIS-29 physiological and psychological sub scores, were associated with higher risk of being a poor sleeper (p<0.001).

**Table 3:** Comparisons of clinical and socio-demographic parameters in MS patients categorized as good sleepers versus poor sleepers.

Variable	Good sleepers	Poor sleepers	*p*-value
Age, Mean±SD	43.2±12.0	46.2±10.4	0.27
Gender, n (%)			0.04
Female	9 (22.0)	32 (78.0)	
Male	15 (46.9)	17 (53.1)	
Married, n (%)			0.80
No	11 (35.5)	20 (64.5)	
Yes	13 (31.0)	29 (69.0)	
Antidepressant drugs, n (%)			0.047
No	24 (36.9)	41 (63.1)	
Yes	0 (0.0)	8 (100)	
Immunotherapy, n (%)			0.21
No	17 (39.5)	26 (60.5)	
Yes	7 (23.3)	23 (76.7)	
ESS, n (%)			0.12
0–10	18 (39.1)	28 (60.9)	
Above 10	5 (20.0)	20 (80.0)	
Pain, n (%)			0.02
No	19 (44.2)	24 (55.8)	
Yes	5 (17.2)	24 (82.8)	
Fatigue (FQ), n (%)			0.001
0–4	21 (46.7)	24 (53.3)	
Above 4	2 (8.0)	23 (92.0)	
Depression (BDI II), n (%)			0.01
0–19	23 (39.7)	35 (60.3)	
Above 19	0 (0.0)	10 (100)	
MSIS-29 physiological, Mean±SD	10.5±13.9	34.9±22.1	<0.001
MSIS-29 psychological, Mean±SD	9.4±12.9	38.0±22.6	<0.001

MSIS-29: Multiple Sclerosis Impact Scale. ESS: Excessive daytime sleepiness.

FQ: Fatigue questionnaire, BDI: Beck Depression Inventory.

Multivariate logistical regression analyses showed that female gender, (OR = 6.8, 95% CI: 1.33–35.3), immunotherapy (OR = 4.7, 95% CI: 0.98–23.3) and reduced quality of life (MSIS-29 psychological subscale) (OR = 1.12, 95% CI: 1.05–1.19) were associated with poor sleep (Table 4).

**Table 4:** Independent factors associated with poor sleep in patients with MS.

Variable	Odds Ratio (OR)	95% CI	*p*-value
MSIS-29; psychological subscale	1,12*	1.05–1.19	0.001
Immunotherapy	4.74	0. 98–23.26	0.054
Gender (female)	6.86	1.33–35.25	0.021

MSIS-29 = Multiple Sclerosis Impact Scale-29;

* OR = 1.12 denotes OR for each increasing point achieved in MSIS-29 psychological subscale.

## Discussion

Poor sleep is a common finding in MS patients, and previous studies have indicated a prevalence of 50% in this patient group [24], [25]. In the present survey, we report a higher frequency of MS patients (67.1%) with disturbed sleep. The focus of this study was to disclose potential modifiable clinical and socio-demographic (age, gender, marital status) risk factors associated with sleep disturbance in MS patients. Among demographic variables that were associated with sleep disturbance, female gender was found to be strongly associated with poor sleep. This is in contrast to a previous study that reported no influence of gender on sleep quality among MS patients [1].

Psychiatric disorders, and especially depression, are prevalent in MS and have major impact on quality of life [26].We found a 3- fold higher prevalence of depressive symptoms among patients compared to controls, consistent with the results of a previous study [27]. All patients classified as having moderate depression in our study suffered from poor sleep. Insomnia and depression are closely linked, and appear to have a bidirectional relationship where insomnia worsens depression and vice versa [28], [29]. MS patients reporting poor sleep should therefore be screened for depression, and treated accordingly.

Subjective sleep disorders in MS and in the general population have in numerous epidemiological studies showed a close a close association with symptoms of fatigue [1], [30], [31]. Sleep disturbance has been reported as a significant independent contributor to fatigue in MS, and in our study, as many as 92% of the patients suffering from fatigue was classified as poor sleepers. A recent randomized controlled therapy study revealed improvement in fatigue by treatment of sleep disorders in MS [32]. However, the pathophysiology of MS-related fatigue is poorly understood and the cause and effect relationship between disturbed sleep and MS- related fatigue is currently an enigma.

Poor quality of sleep in MS patients was not associated with excessive daytime sleepiness, despite increased frequencies of fatigue and sleep complaints in patients categorized as poor sleepers. These results are compatible with a pupillographic and questionnaire (ESS,SSS) study of 61 MS patients, that reported no evidence of excessive daytime sleepiness in MS [33]. Symptoms of fatigue and sleepiness in MS patients are often confused and used interchangeably [34]. Subjective sleepiness and subjective fatigue in MS patients and healthy controls have shown to be independently associated with sleep disorders, although correlated with each other [34]. Patients with insomnia often complain of fatigue, but not sleepiness or the propensity to fall asleep [34]. These studies suggest that fatigue and excessive daytime sleepiness are differentiated conditions and should be assessed independently in patients complaining of poor sleep.

Acute and chronic pain is a frequent symptom in MS, and include neuropathic pain and musculoskeletal pain secondary to other MS symptoms such as spasticity [35]. Population studies have estimated that 55–65% of MS patients suffer from acute, subacute or chronic pain syndrome [36]. We found that the presence of pain was reported about twice as frequent among MS-patients compared to controls. Pain was also significantly more frequent in poor sleepers than in good sleepers. Similar findings was reported in a previous study, that in addition found pain to be the main cause of initial insomnia [37]. In an epidemiologic study of women with RRMS, sleep disturbance intensity were higher for women with RRMS with pain than for women without pain [38]. Pain is a common, but often inadequately treated symptom in MS [39]. By modifying pain, improved sleep may be obtained.

Separate analyses of each PSQI component showed that increased sleep latency was the most frequent complaint among MS patients, followed by sleep disturbance and daytime dysfunction. This was in line with a previous study, where initial insomnia was rated as the most common complaint of the PSQI component values [1].

No significant differences were noted between patients and controls regarding sleep disturbance and sleep efficiency.

The results from our study reveal that the psychological and physiological impact of MS is greater among poor sleepers than among good sleepers. In our study a high psychological burden of MS was independently associated with poor sleep. Nearly half of MS patients have reported significant anxiety levels within the first year of diagnosis [40]. Traditionally, management of MS have focused primarily on treatment of motor symptoms [41]. Treatment of non-motor symptoms such as psychological impact of MS is usually associated with improvement of HRQoL [41]. Sleep disruption evident by polygraphic registration is associated with greater psychological impact on quality of life [42]. More emphasis on the relief of psychological distress of MS may likely favour good sleep.

In this study, the use of immunotherapy was independently associated with poor quality of sleep. An actigraphy study of relapsing-remitting MS patients using immunomodulatory (interferon beta, glatirmaer acetate) drugs, showed a reduction in sleep efficiency in two-thirds on the night following interferon beta injection, irrespective of the frequency of injections [43]. Another study recently reported reduced flu-like symptoms and improved sleep efficacy by switching from evening to morning injections of interferon beta [44].

It is a usual recommendation from neurologists, that interferon beta should be injected in the evening, so that the patient can sleep through the side-effects [45]. We have systematically advised patients to take evening injections of IFNB. Although our results reveal no causality between use of immunotherapy and poor sleep, we believe however, that our findings are sufficiently suggestive to introduce a possible theory on the relationship between timing of immunomodulatory drugs and impact on sleep. The possibility that improved sleep may be obtained by changing administration time of immunomodulatory medication, is important in regards to sleep management, and further studies should be warranted in this regard.

There are several limitations in our study. As this is a cross sectional study, we cannot determine the directions of the predictors of poor sleep. We can only show significant clinical and demographic associations to poor sleep quality. The lower response rate in the control group can be explained by the collection of information by mail, and by a set of questionnaires that was comprehensive and time demanding. Controls suffering from poor sleep may have had particular interest in this subject; this may have skewed the data towards smaller differences between patients and controls.

Micturition-related symptoms such as urge and nocturia were not analysed separately, as these parameters were included in the sleep quality component of MSIS -29 and PSQI respectively. Furthermore, the pain assessment was limited and multidimensional measures such as McGill Pain Questionnaire or Multidimensional pain inventory, would possibly have added more information. The expanded disability scale (EDSS) was not assessed, and therefore a possible relationship between disability status and sleep disturbance could not be established.

The results of this study indicate that poor sleep is a frequent complaint among patients with MS and independently associated with female gender, use of immunotherapy and a high psychological burden of MS. Sleep disturbance is a potentially treatable condition. Increased awareness of modifiable risk factors of poor sleep in MS may be important for early therapeutic and prophylactic interventions.
